# Dental Caries Detection and Classification in CBCT Images Using Deep Learning

**DOI:** 10.1016/j.identj.2023.10.003

**Published:** 2023-11-07

**Authors:** Rasool Esmaeilyfard, Haniyeh Bonyadifard, Maryam Paknahad

**Affiliations:** a Department of Computer Engineering and Information Technology, Shiraz University of Technology, Shiraz, Iran; b Oral, and Dental Disease Research Center, Oral and Maxillofacial Radiology, School of Dentistry, Shiraz University of Medical Sciences, Shiraz, Iran

**Keywords:** Dental caries, Cone beam computed tomography, Artificial intelligence, Deep learning

## Abstract

**Objectives:**

This study aimed to investigate the accuracy of deep learning algorithms to diagnose tooth caries and classify the extension and location of dental caries in cone beam computed tomography (CBCT) images. To the best of our knowledge, this is the first study to evaluate the application of deep learning for dental caries in CBCT images.

**Methods:**

The CBCT image dataset comprised 382 molar teeth with caries and 403 noncarious molar cases. The dataset was divided into a development set for training and validation and test set. Three images were obtained for each case, including axial, sagittal, and coronal. The test dataset was provided to a multiple-input convolutional neural network (CNN). The network made predictions regarding the presence or absence of dental decay and classified the lesions according to their depths and types for the provided samples. Accuracy, sensitivity, specificity, and F1 score values were measured for dental caries detection and classification.

**Results:**

The diagnostic accuracy, sensitivity, specificity, and F1 score for caries detection in carious molar teeth were 95.3%, 92.1%, 96.3%, and 93.2%, respectively, and for noncarious molar teeth were 94.8%, 94.3%, 95.8%, and 94.6%. The CNN network showed high sensitivity, specificity, and accuracy in classifying caries extensions and locations.

**Conclusions:**

This research demonstrates that deep learning models can accurately identify dental caries and classify their depths and types with high accuracy, sensitivity, and specificity. The successful application of deep learning in this field will undoubtedly assist dental practitioners and patients in improving diagnostic and treatment planning in dentistry.

**Clinical significance:**

: This study showed that deep learning can accurately detect and classify dental caries. Deep learning can provide dental caries detection accurately. Considering the shortage of dentists in certain areas, using CNNs can lead to broader geographic coverage in detecting dental caries.

## Introduction

Dental caries is the most common dental disease that, if left uncontrolled, can have serious consequences.^1^ Various methods are available for diagnosing dental caries. Sometimes, the extension of caries is so small that they cannot be visualised or diagnosed without the assistance of radiographic images. X-rays are one of the best methods for diagnosing dental caries and damage to the tooth root.^2^ Cone beam computed tomography (CBCT) plays a significant role in diagnosing dental conditions such as dental caries.^3^ CBCT offers numerous advantages, including multiplanar reconstruction, high accuracy, and low exposure time.^4^^,^^5^ The detection of caries lesions through conventional radiographs remains instead an elusive process. The limitations inherent in traditional radiography are mainly due to the 2D representation of caries lesions, which are 3D structures in reality, and this might lead to the loss of valuable information.

Moreover, the radiographic appearance of a lesion can change dramatically as a function of the chosen projection geometry. The film replacement by digital detectors does not address these fundamental limitations.^6^ Some previous studies showed that CBCT is a superior imaging modality to intraoral digital imaging systems.^7^, ^8^, ^9^

Convolutional neural networks (CNNs) are a type of deep learning model based on neural networks in artificial intelligence that are used for image classification and object detection in images.^10^, ^11^, ^12^, ^13^ CNNs can detect dental caries and analyse dental images to identify caries regions.^14^ They can identify dental caries signs in images, enabling early caries detection using trained neural networks.^15^, ^16^, ^17^, ^18^, ^19^

Deep learning has been widely used in CBCT images.^20^^,^^21^ This technique is used in a variety of fields, including segmentation of the upper airway,^22^, ^23^, ^24^ segmentation of the inferior alveolar nerve,^25^^,^^26^ bone-related disease,^27^^,^^28^ tooth segmentation and endodontics,^29^ temporomandibular joint and sinus disease,^30^^,^^31^ dental implant,^32^^,^^33^ and landmark localisation.^34^^,^^35^ Previous studies evaluated the caries detection performance of deep learning methods. However, there are few studies on neural networks’ performance in caries lesions with different radiographic depths and locations. This is important to health economic perspectives and treatment decision-making, since dental caries treatments, such as remineralisation, cavity filling, root canal therapy, and tooth extraction, vary with lesion depth and location.^14^^,^^36^, ^37^, ^38^

To the best of our knowledge, no study has investigated caries lesions segmentation and classification in CBCT images. Therefore, this study aimed to investigate the accuracy of using deep learning algorithms to diagnose and classify tooth decay according to their extension and location in CBCT images.

## Material and methods

**Fig. 1:**
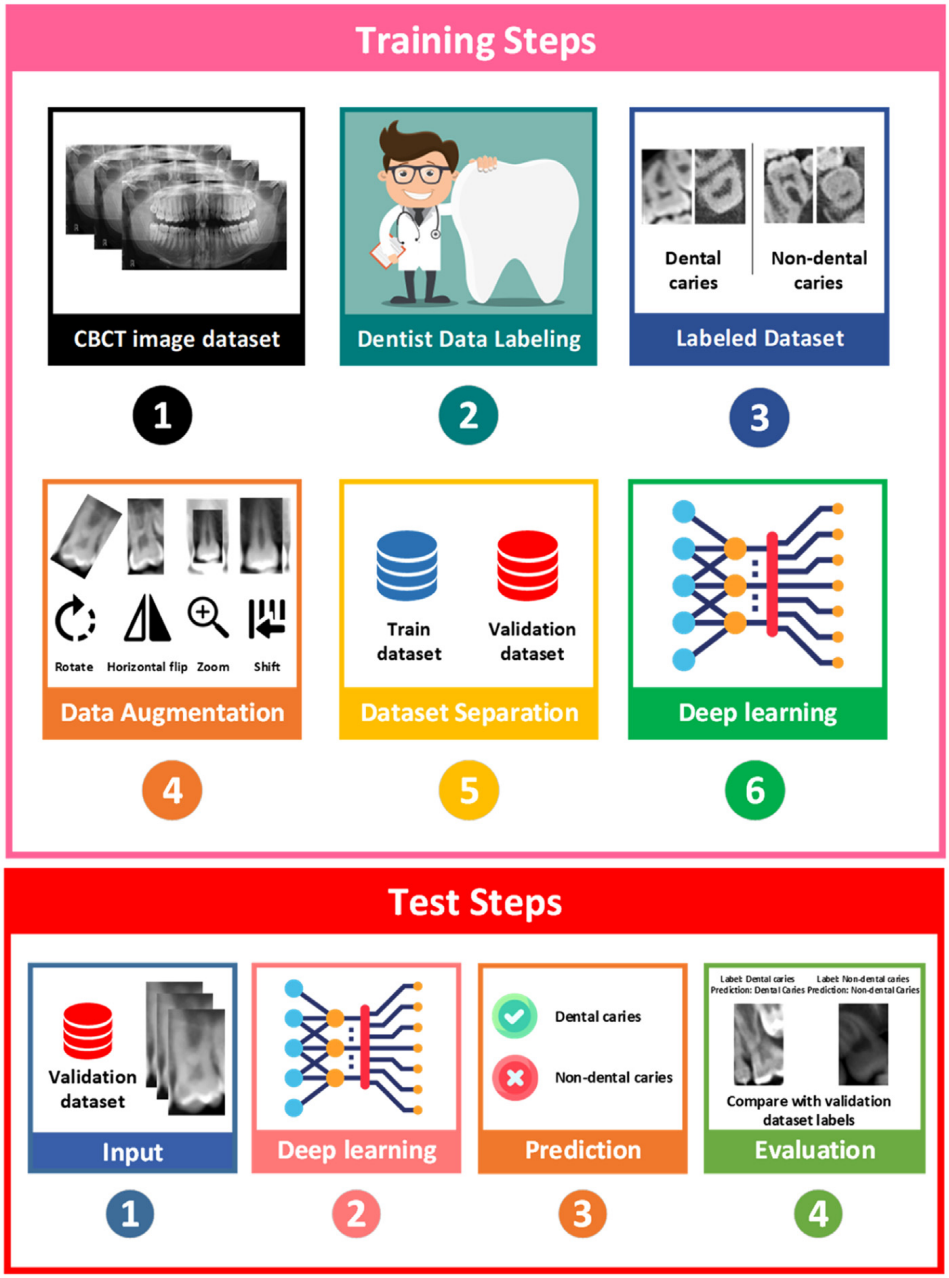
Training and test steps.

The steps of doing the work, training the deep learning model, and then testing this model are shown in Figure 1.

### Dataset collection

The institutional ethics committee approved the present research. An anonymised dataset of CBCT images collected between January 2020 and December 2023 was used in this study. The CBCT images were taken for purposes such as implant placement or evaluation of impacted teeth or paranasal sinuses. The exclusion criterion was poor radiographic image quality, such as severe distortion, blur, and noise. CBCT images were taken using a NewTom VGi scanner (VGi EVO NewTom, Italy) by the following parameters: scanning time 8.9 s, 5 mA, 19 mAs, 120 kV, and reviewed with NNT viewer software. The images were used unidentified.

### Data preparation

In total, 819 CBCT images were labelled by 2 oral and maxillofacial radiologists with 5 years of experience to create the reference standard. The radiologists evaluated all samples and segmented the images by cropping the full images into a rectangular box that fits the teeth. For each teeth, 3 images were evaluated including axial, sagittal, and coronal. These images were cropped only to depict a single tooth in each image. The images were categorised as dental and nondental caries cases. For a single image, a consensus of the expert radiologists was required to determine the final label. In cases of instability, the relevant images were excluded from the study. The final dataset consisted of 785 molar teeth images, of which 382 were carious and 403 were noncarious cases. The carious teeth were divided into 4 types based on the location of caries. Occlusal caries detected on the CBCT radiographs were labelled as type I, proximal caries as type II, caries in the cervical region as type III, and teeth with more than 1 caries as type IV. As a result, there were 99 occlusal caries (type I), 107 proximal caries (type II), 57 cervical caries (type III), and 119 cases of more than 1 caries (Type IV). For classification of the caries extension, there were the following 3 classes: “D1” caries had radiolucency in enamel or the outer third of dentin; “D2” caries had radiolucency in the middle third of dentin; and “D3” caries had radiolucency in the inner third of dentin with or without apparent pulp involvement. All annotated areas on an image ultimately constructed the reference dataset, which consists of 110 D1 lesions, 140 D2 lesions, and 132 D3 lesions.

Subsequently, a data augmentation procedure was applied to increase the training data volume. In this regard, all tooth images were vertically and horizontally flipped. Additionally, the dataset was augmented through random rotations (20°), and the images were magnified up to a maximum of 2 times.^39^ In the present study, data augmentation increased the number of images about 10 times. The dataset ultimately consisted of 7850 cases, including 3820 dental caries cases and 4030 nondental caries cases, all converted to PNG format. The images were resized to dimensions of 96 × 160 pixels. Seventy percent of the data were used as the training dataset, and the remaining 30% were allocated as the test dataset. The selection of data for these datasets was done randomly. The number of dental and nondental caries samples increased to 5495 for the training steps. This dataset includes 2674 dental caries images and 2821 nondental caries images.The flowchart of the data extraction procedure is shown in Figure A1 Appendix. Finally, the data were provided as input to the deep convolutional neural network algorithm, and this network was trained.

### The multiple-input deep convolutional neural network architecture

**Fig. 2:**
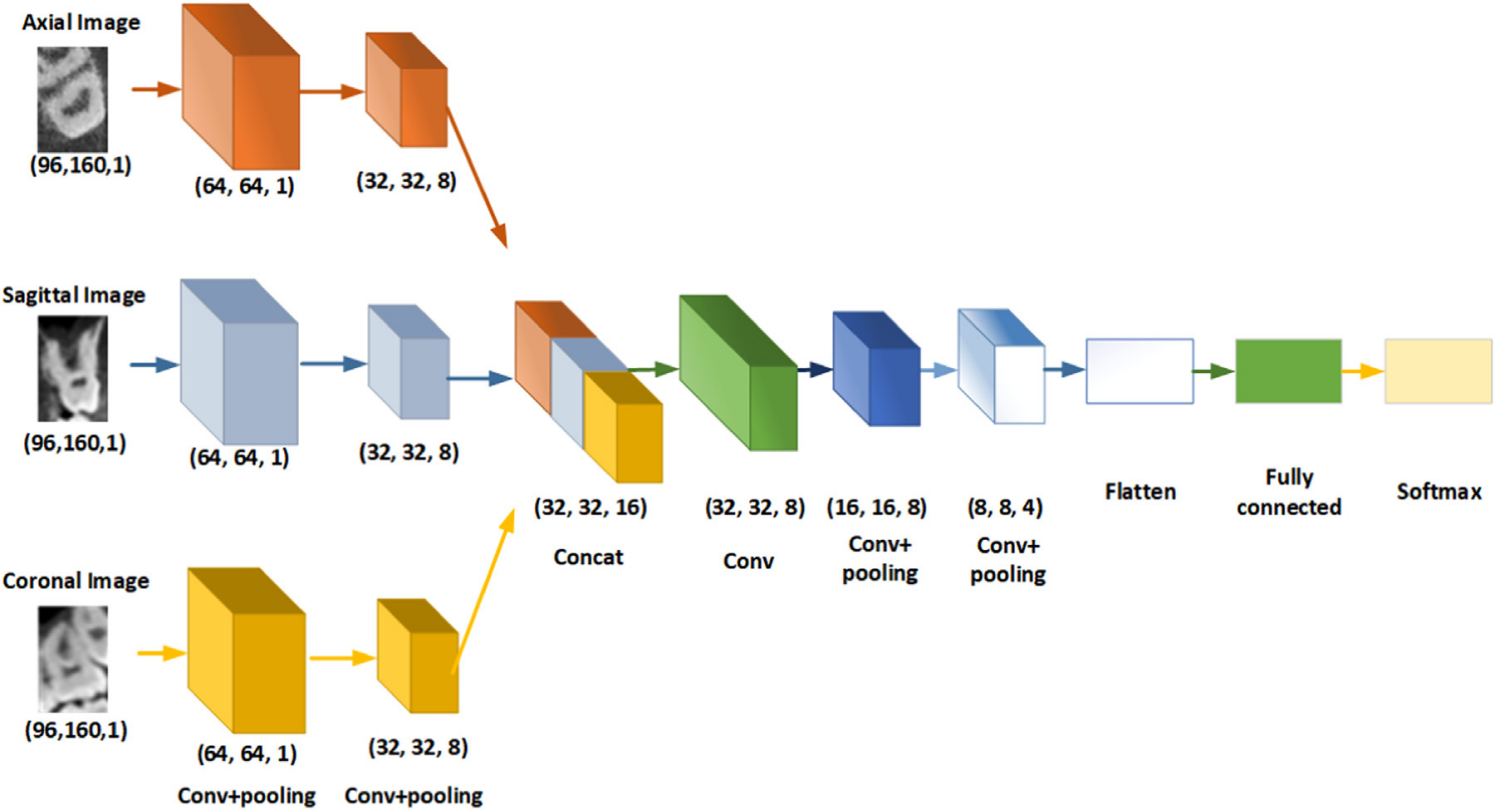
The multiple-input deep convolutional neural network architecture.

Figure 2 shows a view of the proposed multiple-input deep convolutional neural network architecture. The network structure starts with 3 inputs for 3 input images of axial, sagittal, and coronal. The image is resized and normalised in several layers of convolution and pooling. Then, these 3 inputs are combined and concatenated again through several steps, and by repeating this operation, they are flattened into a vector. In the final part, the input vector is entered into a fully connected deep network to perform the classification operation.

Based on the presented architecture, the classification was done in 3 steps. First, the images were presented as input to this network and categorised based on dental and nondental caries. Second, the cases identified as dental caries were presented to this architecture in 2 separate steps and were categorised once based on type and once based on location.

### Configuration and evaluation metrics

This model was implemented using the PyTorch library in the Python programming language.The implementation was carried out in the Google Colab environment.^36^ The training dataset was randomly divided into 32 batches per epoch, and the model was trained for 100 epochs with a learning rate of 0.001. A fine-tuning of parameters was performed using the BCEWithLogitsLoss cost function, the Adam optimisation algorithm, and the CosineAnnealingLR learning rate scheduler to improve the detection of dental caries. The weights with the highest values were used when the validation accuracy did not improve.

After training the CNN network, the test dataset was provided to the neural network, and the network made predictions regarding the presence or absence of dental caries for the provided samples. The results of these predictions were compared and evaluated against the labels of the test dataset. The accuracy, sensitivity, specificity, and F1-score metrics were examined during the evaluation.

## Results

The reliability of the interexaminers applying κ statistics was assesed. The images were evaluated again for checking the consistency between the first and the second sets of records. Acceptable interexaminer agreement (kappa values >0.75) was detected.

The accuracy, sensitivity, specificity, and F1-score on test dataset for nondental caries cases were 94.8%, 94.3%, 95.8%, and 94.6%. The results of these metrics on the same dataset for dental caries cases were 95.3%, 92.1%, 96.3%, and 93.2%. The result of classification for different types and extensions are presented in Table 1 and Table 2. The methods yielded accuracy, sensitivity, specificity, and F1-score of more than 80% for all types of dental caries. Type III showed lower results than other types.

**Table 1:** Result of dental caries classification for different types.

Type	Accuracy	Sensitivity	Specificity	F1-Score
I (Occlusal)	93.3%	92.3%	94.6%	92.8%
II (Proximal)	93.7%	95.2%	94.2%	94.4%
III (Cervical)	91.6%	88.7%	91.3%	89.4%
IV (More than one caries)	97.2%	95.1%	96.4%	94.0%

**Table 2:** Result of dental caries classification for different extensions.

Extension	Accuracy	Sensitivity	Specificity	F1-Score
D1	89.7%	87.7%	90.6%	89.0%
D2	93.3%	95.0%	94.2%	93.6%
D3	96.2%	96.5%	97.5%	97.3%

**Fig. 3:**
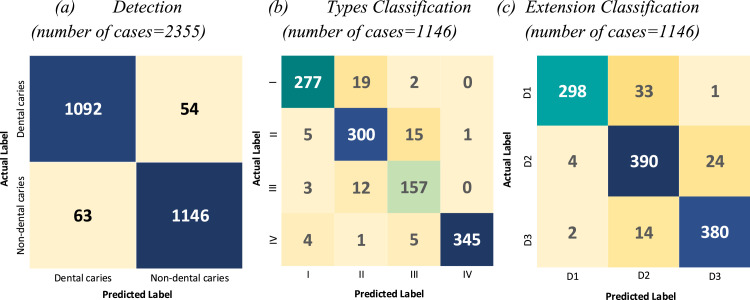
Confusion matrices.

The results given in Figure 3 show that the proposed models produced effective results for type I, type II, and type IV caries detection. However, the proposed network architecture for detecting type III caries remained weak, possibly due to insufficient datasets of this type. The model showed better results in detecting D3 lesions. The accuracy of the predicted labels for the detection and classification of types and extensions in the test datasets is visualised as confusion matrices in Figure 3.

## Discussion

This research investigated the accuracy of deep learning algorithms and machine learning approaches for detecting the extension and location of dental caries in CBCT images. The results of this study demonstrated that deep learning is a precise and highly efficient tool for detecting dental caries. Similarly, most previous studies showed relatively high precision, sensitivity, and specificity in deep learning for detecting caries (usually more than 80%). In this study, oral and maxillofacial radiologists were considered as the gold standard.

Various studies have compared the accuracy, sensitivity, and specificity of using CNN networks and deep learning to detect dental caries in dental radiographs (Table A1 Appendix). Previous studies have obtained an accuracy of CNN networks for caries detection ranging from 82% to 97%. The differences in results can be attributed to various factors, such as the use of different datasets. Datasets can vary in size, diversity, data quality, and distribution. The selection of layers, types of layers, number of neurons, learning rate, and other model parameters can also significantly impact the results. Machine learning techniques, optimisation algorithms, and other methods can influence the model's performance and accuracy. Variations in preprocessing methods and data preprocessing procedures can also lead to differences in the results. Our study achieved an accuracy of more than 94.6%. The accuracy of our model was higher than that of models in previous studies and yielded a score of 0.986. The study by Geetha et al^40^ shows the highest accuracy of all studies. However, this study is a special case because it uses a very shallow neural network and only a hidden layer to build its feature extractor, which is now rare. The authors of this study used only 105 images and 10-fold cross-validation, so their model was not evaluated on a hold test set.

The sensitivity and specificity of previous studies were between 63.29%−92.3% and 60.71%−100%, respectively. Our study's sensitivity was 94.5% and specificity was 91.8%, which was better and more desirable than previous studies (Table A1 in Appendix). The results of this study are higher than previous studies and can be examined from two perspectives. First, this study used 3D CBCT images of higher quality. In comparison, panoramic and periapical images with lower quality were used in previous studies. In addition, using multiple-input architecture and data augmentation has sought high accuracy in previous image studies. This difference in measurement could be due to the technique used.^41^

In the present study, the accuracy of the classification of the location of the dental caries was higher than in the study of Day et al,^42^ especially for cervical caries. The possible reason may be the use of panoramic radiographs in that study. Panoramic radiographs are 2D images, and this type of caries is usually located in the vestibule and lingual of the teeth. Hence, it overlaps with the denser healthy tooth tissue, and, at the same time, it can be challenging to detect because of overlapping with the radiolucent reflection of the pulp chamber on the radiograph.

The results of this study indicated that deep learning could automatically learn the differences amongst caries depths in caries extension image features and achieve effective interpretations, especially for D3 lesions similar to the study of Lian et al.^43^ The possible reason is that D3 lesions have a more extensive range of transmission images in panoramic films and are easier to detect with the naked eye. Caries in the D1 and D2 stages are more likely to be missed or have lesion boundaries that are difficult to determine.

Prados-Privado et al^44^ have presented a systematic review on caries diagnosis and detection using neural networks and concluded that the best results were obtained in the study using only one examiner, so the same criteria were continuously used in caries detection and then in the study in which 4 experts analysed the images. Finally, the worst results in terms of accuracy were obtained by studying with 2 examiners. Therefore, only a single examiner was used in this study.

CBCT has many advantages that aid in dental caries detection. The high accuracy of this technology allows for the detection of small cavities. The imaging of CBCT has fewer geometric distortion problems than conventional methods in theory; thus, the real multiplanar reformation of caries lesions was available, which was impossible before. Many incipient carious lesions remain undetected on intraoral radiographs due to inappropriate image angulation or overlap of contact areas.^45^ CBCT systems were superior to conventional methods in detecting occlusal caries. The superior detection of recurrent or residual caries using CBCT may be due to multiplanar images produced by the CBCT (axial, coronal, and sagittal planes).^46^^,^^47^

Recent studies have shown that CNNs can accurately detect dental caries. CNNs can provide dental caries detection at much lower costs than dental examinations. Considering the shortage of dentists in certain areas, using CNNs can lead to broader geographic coverage in detecting dental caries. However, to ensure the accuracy of dental caries diagnosis, it may still be necessary to have a dental professional review the results. Furthermore, using CNNs for dental caries detection requires accurate translation of radiographic data and training the networks with sufficient and diverse datasets.

This method still requires further refinement and improvement. Additionally, this approach requires more complex equipment and powerful processing capabilities, which may not be readily available in some dental centers. In addition, the images used during the training process must be labeled by experts. Therefore, when artificial intelligence is trained using the scores of human observers, the system cannot exceed the trainer. Thus, performance depends on the quality of input. Expert judgments are usually used as standard guidelines. It is important to stress that manual marking by experts provides the reference needed to train and assess models but does not necessarily represent the truth. The use of a histologic gold standard method is essential for the validation of a method of caries diagnosis. We applied no gold standard in the study, such as microcomputed tomography and histology of extracted teeth. However, dentists with different experiences and professional backgrounds are required for comparison, which may provide more valuable information. Second, labelling in the constructed reference test was not sufficiently precise, as it was not triangulated with the gold standard (histology). None of the studies included in the present review referenced the standard used. Future studies would likely pave the way for applications of such AI systems in dental caries detection and, in a greater aspect, prevention and control of dental caries in communities.

## Conclusion

This study demonstrated that deep learning models can accurately identify dental caries with high accuracy, sensitivity, and specificity. This study highlights the potential power of deep learning in detecting dental caries using CBCT images. The successful application of deep learning models in this field will undoubtedly assist dental practitioners and patients in improving diagnostic and treatment planning in dentistry.

## Conflict of interest

None disclosed.

## Appendix Supplementary materials

Image, application 1

## Footnotes

Supplementary material associated with this article can be found in the online version at doi:10.1016/j.identj.2023.10.003.
