# Supplementary material for: Dental Caries Detection and Classification in CBCT Images Using Deep Learning
Source: Int Dent J. 2023 Nov 7;74(2):328–34. doi: 10.1016/j.identj.2023.10.003 (PMC10988262; doi:10.1016/j.identj.2023.10.003)
Supplement: Supplementary file 1 [file mmc1.docx]

**Appendix**


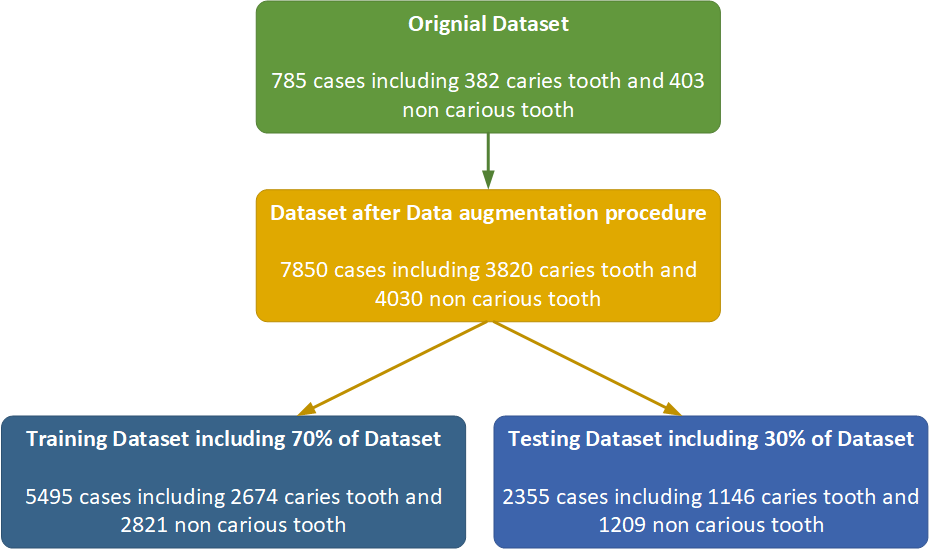


Figure A1. Flowchart of data extraction

Table A1. Comparison of Previous Studies in Dental Caries Detection

| F1-Score | Specificity | Sensitivity | Accuracy | Technique | Image Type |  |
| --- | --- | --- | --- | --- | --- | --- |
| 93.2% | 96.3% | 92.1% | 95.3% | Multiple-input CNN | CBCT | Ours |
| - | 83-94 % | 81-92.3% | 82-88% | GoogLeNet Inception v3 | Periapical | Lee, et al. [15] |
| 64.14% | - | 63.29% | - | U‑Net | Bitewing | Lee, et al. [45] |
| 86% | 88% | 86% | 87% | MobileNet V2 | Panorama | Vinayahalingam, et al. [38] |
| 73% | 83 % | 75 % | 80% | U-Net  Intersection-over-Union | Bitewing | Cantu, et al. [39] |
| 95.2% | - | 95.2% | 97.1% | FFBPNN with one hidden layer | Periapical | Geetha, et al. [41] |
| 30% | 60.71% | 77.87% | 70% | ResNet | Bitewing | Panyarak, et al. [46] |
| 83.7% | 85.2% | 86.9% | 85.9% | CNN trained with Edge Extraction | Periapical | Lin, et al. [47] |
| - | 88.7% | 74% | 81.3% | U‑Net | Intraoral photograph | Park, et al. [48] |
| 69% | - | 65.2% | 83.2% | nnU-Net  DenseNet121 | Panorama | Lian, et al. [44] |
